# Long-term variability of impulse oscillometry and spirometry in stable COPD and asthma

**DOI:** 10.1186/s12931-022-02185-5

**Published:** 2022-09-21

**Authors:** Jianhua Xu, Xingxing Sun, Hanqing Zhu, Yuan Cao, Bigyan Pudasaini, Wenlan Yang, Jinming Liu, Jian Guo

**Affiliations:** 1 Department of Pulmonary Function Test, Shanghai Pulmonary Hospital, School of Medicine, Tongji University, Shanghai, 200092 China; 2 Department of Internal Medicine, Columbia Bainuo Clinic, Shanghai, 200040 China; 3 Department of Pulmonary Circulation, Shanghai Pulmonary Hospital, School of Medicine, Tongji University, Shanghai, 200092 China

**Keywords:** IOS, Variability, COPD, Asthma

## Abstract

**Background:**

While optimizing spirometry is a challenge for lung function labs, long-term variability if any between IOS (impulse oscillometry) parameters and spirometry is not clearly known in stable COPD (chronic obstructive pulmonary disease) and chronic asthma. The forced oscillation technique is increasingly employed in routine lung function testing. Our aim in this study was to determine the variability in oscillometric parameters between clinic visits over weeks or months in two patient groups during a period of clinical stability. Moreover, the research assessed relationships between IOS parameters long-term variability and COPD severity.

**Methods:**

We used data from 73 patients with stable COPD and 119 patients with stable asthma at the Shanghai Pulmonary Hospital Affiliated to Tongji University. Patients were included if they had three or more clinic visits where spirometry and IOS were performed during a clinically stable period. Data recorded from the first three visits were used. The standard deviation (SDbv), the coefficient of variation (COV), intraclass correlation coefficient (ICC) and the coefficient of repeatability (COR) were calculated, Wilcoxon Mann–Whitney test was used for data that did not conform to normality of distributions, Kruskal Wallis test was used to compare with multiple groups, post hoc comparison was analyzed by Bonferroni, Spearman correlation coefficients for non-parametric data, the multiple regression analyses to determine the relationship between long-term variability and airflow obstruction.

**Results:**

(1) The repeatability of IOS resistance parameters with ICC values > 0.8 was high in COPD and asthma. ICC values of IOS resistance parameters were higher than IOS reactance parameters; (2) the repeatability of spirometry parameters with ICC values < 0.8 was lower than IOS resistance parameters in different GOLD (the Global Initiative for Chronic Obstructive Lung Disease) stages, the higher the stage the worse the repeatability; (3) the severity of airflow obstruction was correlated with long-term variability of R5 (R at 5 Hz) (P < 0.05) in GOLD4, not with long-term variability of R20 (R at 20 Hz) (P > 0.05) and R5-R20 (P > 0.05).

**Conclusion:**

IOS resistance parameters have good long-term repeatability in asthma and COPD. Additionally, repeatability of spirometry parameters is lower than IOS resistance parameters in different GOLD stages.

## Introduction

IOS is increasingly receiving attention for pulmonary function examinations. The main advantage being its simplicity: the oscillatory signal is based on spontaneous breathing and, hence, no special patient cooperation is required [1].

IOS is a variant of forced oscillation technique (FOT), measures respiratory impedance at multiple frequencies in a range of 5–35 Hz. Measurements are performed at tidal breathing. The principle is to measure the flow response of the respiratory tract to externally applied pressure signals. Hence, the respiratory impedance can be derived, which is expressed in its component resistance and reactance [2]. The resistance at 5 Hz (R5) represents the total airway resistance, the resistance at 20 Hz (R20) indicates the resistance of the large airways, (R5–R20) reflects resistance in the small airways, these three parameters are commonly used to evaluate airway resistance [3]. Chronic airway obstruction is a major symptom feature of COPD and asthma, forced expiratory volume in 1 s (FEV1) is the gold standard for diagnosing airway obstruction. However, FEV1 is thought to be inadequate to determine obstruction of the smaller airways [4, 5]. Some studies suggest that IOS is more sensitive than spirometry for detecting airway obstruction in patients with asthma and COPD [1, 6, 7]. Therefore, IOS is a useful complimentary method in detecting small airway obstruction.

IOS as a re-emerging clinical detection technology is valuable to study the minimal clinically important difference (MCID) of lung function in stable COPD and asthma. There has been suggestion that the ICC’s are high for all parameters in health, stable asthma and COPD. FOT measures are highly repeatable, day-to-day variability is due mostly to repeatability, which is correlated with airway obstruction [8]. The between-visit variability of a range of IOS parameters in a group of patients with asthma in the stable state, and between-visit variability over two-time intervals, namely in 2 weeks and 3 months have been studied previously and ICC values were > 0.8 in majority of the cases. Therefore, suggesting IOS parameters are stable over time and have the potential to be employed for clinical testing in asthma [9]. Moreover, FOT parameters have good long-term repeatability with high ICC values in health, asthma and COPD, but also that variability differs between diseases [10]. While the short-term variability in IOS parameters is known, longer-term variability still needs more work. Understanding their variations in clinically stable patients on routine outpatient visits is necessary to estimate clinically important changes with time.

Therefore, the aim of this study was to determine the variability in IOS parameters between clinic visits over weeks or months, in stable asthma and COPD patients. The research also assessed relationship between IOS parameters long-term variability and degree of airway obstruction of stable COPD.

## Patients and methods

### Ethical approval

The study protocol was reviewed and approved by the Ethics Committee of Shanghai Pulmonary Hospital. All methods including PFT (pulmonary function tests) and IOS were carried out in accordance with relevant guidelines and regulations.

### Patients

This study retrospectively enrolled 73 patients with stable COPD and 119 patients with stable asthma who were examined in Shanghai Pulmonary Hospital from January 2011 to December 2018. All patients were in the stable condition. The main inclusion criteria were as follows: (1) three or more clinic visits; (2) spirometry and IOS were performed; (3) no change in symptoms, no respiratory infection in the past 6 weeks and no changes in treatment. Patients were diagnosed by physicians as asthma and COPD, having a post-bronchodilator Tiffeneau index FEV1/FVC < 70 (forced vital capacity, FVC) [10]. GOLD staging was conducted for patients with COPD according to the 2018 COPD Guidelines [11].

### PFT

We performed pulmonary function tests [including spirometry and impulse oscillation (IOS)] using standard equipment (Masterscreen-PFT, Jaeger crop, Hoechberg, Germany; Masterscreen-IOS, Jaeger crop, Hoechberg, Germany). FVC, FEV1 and FEV1/FVC were determined by standard procedure [12]. Oscillometry was recorded prior to spirometry. IOS test time was 30 ~ 45 s, being repeated three times with an interval of 1 min, and the best value was selected. IOS parameters recorded were R5, R20, R5-R20, Xrs at 5 Hz (X5), which indicate elastic and volume orient properties of the peripheral lung, resonant frequency (Fres), where Xrs crosses zero and the elastic and inertial forces are equal in magnitude and opposite, and a low-frequency reactance area (AX), which is an integral of X5 to Fres. Spirometry was measured three times and the optimal curve was selected, as per the standard recommended by The European Respiratory Society [13].

### Statistical analysis

Statistical analysis was performed using SPSS 22.0 and GraphPad Prism 5. Data are shown as mean ± SD, median (interquartile range). A two-tailed P-values less than 0.05 is considered to be statistically significant. The Shapiro–Wilk test was used to assess normality of distributions. Long-term variability was expressed as the standard deviation of the first three visit’s measurements for each subject (SDbv). The coefficient of variation (COV = SDbv/mean) was calculated and intraclass correlation coefficient (ICC; mixed-effects model, absolute agreement, mean of three raters) of IOS measurements of the three clinic visits. In addition, the coefficient of repeatability (COR) was calculated, defined as twice the standard deviation of the differences between two pairs of consecutive clinic visits from three clinical visits per patient or expressed as a percentage of close to maximal variation (pMV) [14, 15]. ICC value between 0.5 and 0.6 was considered as medium repeatability, between 0.7 and 0.8 was considered as good repeatability, and > 0.8 was considered as very good repeatability. pMV between 0 and 33% was considered decent repeatability, between 33 and 66% was considered good repeatability, and above 66% was considered poor repeatability. Age and BMI (body mass index) were analyzed by one-way ANOVA. Wilcoxon Mann–Whitney test was used for data that did not conform to normality of distributions, Kruskal Wallis test was used to compare with multiple groups, post hoc comparison was analyzed by Bonferroni. The relationships between variability (SDbv) and %FEV1 (FEV1 as a percentage of predicted) were examined using Spearman correlation coefficients for non-parametric data. Multiple regression analysis was used to analyze the factors that might influence the variability of IOS parameters in stable COPD patients between long-term clinic visits.

## Results

### Baseline demographic characteristics and lung function

The demographic data and the baseline lung function test results of the COPD and asthma patients are shown in Table 1. 119 asthma and 73 COPD patients (n = 1 Global Initiative for Chronic Obstructive Lung Disease (GOLD) stage 1; n = 22 GOLD stage 2; n = 31 GOLD stage 3; and n = 19 GOLD stage 4) were included. Since there was only one GOLD stage 1 patient, GOLD stage 1 and GOLD Stage 2 were combined for subsequent analysis. Asthma [(49.86 ± 14.26) years] patients were younger than COPD [(62.68 ± 9.57) years] patients (F = 45.08, P < 0.0001), and there was no statistically significant difference in BMI between the two groups (F = 0.93, P = 0.337). There were no statistically significant differences in age and BMI among COPD GOLD1-2, GOLD3 and GOLD4 stage groups (F = 0.58, P = 0.562; F = 1.92, P = 0.155). Statistically significant differences in spirometry parameters between COPD and asthma (P < 0.0001) was noted, the spirometry parameters in COPD were lower than asthma. There were statistically significant differences in IOS parameters, except for R20 (P = 0.778) and Freq (P = 0.507), the COPD patients had higher R5, R5-R20, AX and more negative X5 compared to the asthma group (P < 0.0001).

**Table 1:** Baseline characteristics and lung function of COPD and asthma

	COPD-All (N = 73)	COPD-GOLD1-2 (N = 23)	COPD-GOLD3 (N = 31)	COPD-GOLD4 (N = 19)	Asthma (N = 119)	P value
Age, year	62.68 ± 9.57	63.04 ± 10.19	63.65 ± 9.20	60.68 ± 9.62	49.86 ± 14.26	< 0.0001
M3-M1, month	6.1 (3.4–16.6)	6.1 (3.3–22.8)	8.4 (3.0–19.0)	4.9 (4.0–12.6)	4.9 (4.0–12.6)	
BMI, kg/m^2^	22.81 ± 3.59	23.85 ± 3.60	22.72 ± 3.58	21.71 ± 3.41	23.48 ± 3.48	0.337
FVC, L	2.02 (1.66–2.65)	2.91 (2.07–3.50)	2.02 (1.58–2.47)	1.74 (1.57–2.09)	2.88 (2.40–3.73)	< 0.0001
FEV1, L	0.95 (0.72–1.29)	1.51 (1.26–1.87)	0.89 (0.73–1.09)	0.69 (0.57–0.75)	2.36 (1.86–2.82)	< 0.0001
MEF 50, L/s	0.42 (0.30–0.71)	0.90 (0.71–0.94)	0.39 (0.31–0.54)	0.30 (0.22–0.33)	3.17 (2.56–4.03)	< 0.0001
MEF 25, L/s	0.19 (0.14–0.25)	0.29 (0.22–0.35)	0.19 (0.12–0.21)	0.15 (0.11–0.19)	0.85 (0.52–1.17)	< 0.0001
MMEF 75/25, L/s	0.37 (0.26–0.58)	0.67 (0.60–0.80)	0.35 (0.26–0.41)	0.25 (0.20–0.29)	2.16 (1.37–2.89)	< 0.0001
R5, cmH_2_O/(L/s)	5.98 (4.64–7.64)	4.82 (4.06–6.77)	7.23 (5.55–8.51)	5.83 (4.60–7.21)	4.25 (3.13–5.11)	< 0.0001
R20, cmH_2_O/(L/s)	3.17 (2.68–3.79)	2.97 (2.72–3.64)	3.63 (2.86–4.61)	2.69 (2.10–3.48)	3.17 (2.56–4.03)	0.778
R5-R20, cmH_2_O/(L/s)	2.85 (1.89–3.9)	1.73 (0.94–3.01)	3.60 (2.60–4.03)	2.85 (2.48–4.04)	0.85 (0.31–1.59)	< 0.0001
AX, cmH_2_O/L	45.70 (21.87–61.19)	18.76 (8.16–31.44)	52.88 (40.08–72.50)	49.67 (31.88–75.15)	6.91 (3.03–12.99)	< 0.0001
Freq, 1/s	28.8 (22.49–36.34)	21.52 (16.47–27.10)	33.99 (28.47–38.09)	28.8 (25.85–39.99)	16.82 (13.52–22.14)	0.507
X5, cmH_2_O/(L/s)	− 4.57 (− 2.67–6.88)	− 1.89 (− 1.44–3.84)	− 5.82 (− 4.17–8.13)	− 5.99 (− 3.96–7.86)	− 1.44 (− 0.89–1.76)	< 0.0001

The data are presented as mean ± SD, median (interquartile range). Age and BMI were analyzed by one-way ANOVA, post hoc comparison was analyzed by Bonferroni, Wilcoxon Mann–Whitney test was used for data that did not conform to normality of distributions. M3-M1 represents the month difference between the third and first follow-up; COPD = chronic obstructive pulmonary disease; GOLD = the Global Initiative for Chronic Obstructive Lung Disease; BMI = Body Mass Index; FVC = forced vital capacity; FEV1 = forced expiratory volume in 1 s; MEF = maximal expiratory flow; MMEF = maximum midexpiratory flow; R5 = R at 5 Hz; R20 = R at 20 Hz; AX = a low-frequency reactance area; Freq = resonant frequency; X5 = Xrs at 5 Hz; P value COPD versus asthma

### Long-term variability in COPD and Asthma

**Fig. 1:**
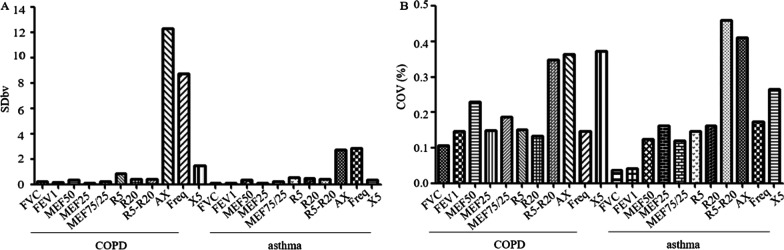
Long-term variability of lung function and IOS parameters in stable COPD and asthma: SDbv (**A**) and COV (**B**) using GraphPad Prism 5 (Graphing replicates or error bars plot: Median). See Table 1 and Table 2 legend for expansion of abbreviations

Long-term variability of spirometry and IOS parameters is shown in Table 2. The relative between-visit variability (SDbv) for IOS was higher than for spirometry. SDbv of FVC, FEV1, MEF50, MEF25, MMEF75/25, R5, R5-R20, AX, Freq and X5 were statistically significantly different between COPD and asthma (Z = − 6.38, P < 0.0001; Z = − 3.64, P < 0.0001; Z = − 8.28, P < 0.0001; Z = − 8.03, P < 0.0001; Z = − 8.50, P < 0.0001; Z = − 3.64, P < 0.0001; Z = − 5.15, P < 0.0001; Z = − 8.18, P < 0.0001; Z = − 7.59, P < 0.0001; Z = − 7.95, P < 0.0001), SDbv of FVC, FEV1, R5, R5-R20, AX, Freq and X5 in COPD were higher compare to asthma. SDbv of R20 were no statistically significantly different between COPD and asthma (Z = − 1.41, P = 0.16). Between-visit variability relative to the mean (COV) of IOS parameters (R5, R20, AX, Freq) were not statistically significantly different between COPD and asthma (Z = − 0.37, P = 0.713; Z = − 1.63, P = 0.102; Z = − 1.12, P = 0.263; Z = − 1.27, P = 0.203). COV of FVC, FEV1, MEF50, MMEF75/25, and X5 in COPD were higher compare to asthma (Fig. 1).

**Table 2:** Long-term variability of spirometry and IOS parameters in COPD and Asthma

	SDbv	COV	ICC	COR
COPD				
FVC, L	0.25 (0.16–0.35) ^a^	11% (6–16%) ^a^	0.96	0.71 (25%)
FEV1, L	0.15 (0.09–0.22) ^a^	15% (8–20%) ^a^	0.94	0.45 (23%)
MEF 50, L/s	0.10 (0.06–0.19) ^a^	20% (10–31%) ^a^	0.94	0.40 (26%)
MEF 25, L/s	0.04 (0.02–0.08) ^a^	18% (11–28%)	0.84	0.18 (45%)
MMEF 75/25, L/s	0.08 (0.05–0.13) ^a^	17% (12–25%) ^a^	0.94	0.31 (28%)
R5, cmH_2_O/(L/s)	0.86 (0.52–1.28) ^a^	15% (10–21%)	0.90	2.73 (35%)
R20, cmH_2_O/(L/s)	0.40 (0.29–0.61)	13% (10–19%)	0.84	1.62 (47%)
R5-R20, cmH_2_O/(L/s)	0.71 (0.45–1.16) ^a^	29% (20–45%) ^a^	0.84	2.54 (46%)
AX, cmH_2_O/L	12.33 (6.88–19.30) ^a^	37% (24–56%)	0.85	44.35 (42%)
Freq, 1/s	3.88 (2.33–6.59) ^a^	15% (9–23%)	0.78	15.05 (50%)
X5, cmH_2_O/(L/s)	1.46 (0.62–2.47) ^a^	37% (22–52%) ^a^	0.78	5.08 (47%)
Asthma				
FVC, L	0.11 (0.06–0.17)	4% (2–6%)	0.99	0.42 (12%)
FEV1, L	0.10 (0.06–0.16)	4% (2–7%)	0.99	0.36 (12%)
MEF 50, L/s	0.33 (0.20–0.48)	12% (8–17%)	0.96	1.24 (23%)
MEF 25, L/s	0.12 (0.08–0.23)	16% (9–27%)	0.95	0.72 (30%)
MMEF 75/25, L/s	0.24 (0.14–0.37)	12% (8–18%)	0.96	1.03 (23%)
R5, cmH_2_O/(L/s)	0.56 (0.33–0.90)	15% (9–21)	0.93	1.94 (36%)
R20, cmH_2_O/(L/s)	0.51 (0.29–0.80)	16% (10–23%)	0.90	1.70 (46%)
R5-R20, cmH_2_O/(L/s)	0.40 (0.22–0.64)	46% (27–68%)	0.83	1.42 (47%)
AX, cmH_2_O/L	2.71 (1.22–4.75)	41% (27–62%)	0.81	17.35 (54%)
Freq, 1/s	2.87 (1.34–4.95)	17% (11–30%)	0.66	16.23 (69%)
X5, cmH_2_O/(L/s)	0.33 (0.18–0.69)	27% (16–46%)	0.62	2.53 (80%)

Wilcoxon Mann–Whitney test was used for data analysis. COR is expressed as absolute (cmH2O·s·L-1) and pMV (%). SDbv = between-visit standard deviation; COV = the coefficient of variation; ICC = intraclass correlation coefficient; COR = the coefficient of repeatability. ^a^P < 0.05 COPD versus asthma. See Table 1 legend for expansion of abbreviations

The COR data during a period of stable disease for the two patient groups (Table 2) are novel. We showed that variations in R5 up to 35% in COPD and 36% in asthma were typical of stable patients. Variations in R20 up to 47% in COPD and 46% asthma can be present, equivalent to R5-R20 up to 46% in COPD and 47% in asthma, the COR values < 66% were considered good repeatability, the COR values of IOS resistance parameters and spirometry parameters were similar between COPD and asthma, and the COR values of IOS reactance parameters in asthma were higher than in COPD. However, the repeatability of spirometry parameters, IOS resistance parameters and AX with high ICC values noted, the repeatability of Freq and X5 with ICC values < 0.8 was low in COPD and asthma. Moreover, the relative CORs of IOS parameters were more variable compare to spirometry, but importantly, the ICCs of IOS resistance parameters were similar.

Comparison of long-term variability of IOS parameters with the degree of airway obstruction in stable COPD.

Long-term variability of PFT and IOS parameters related to COPD severity stages are shown in Table 3. Between-visit variability (SDbv) of FVC, R5, AX and X5 were statistically significantly different among GOLD1-2, GOLD3 and GOLD4 patients (H = 9.34, P = 0.009; H = 8.85, P = 0.012; H = 18.93, P < 0.0001; H = 26.45, P < 0.0001). Especially, SDbv of FVC was statistically significantly different between GOLD1-2 and GOLD4 patients (P < 0.05). SDbv of R5 was statistically significantly different in GOLD3 and GOLD4 patients (P < 0.05), SDbv of AX and X5 were statistically significantly different between GOLD1-2 and GOLD3, and GOLD1-2 and GOLD4 (P < 0.05). Between-visit variability relative to the mean (COV) for FVC, FEV1, R5, R5-R20 and X5 were statistically significantly different among GOLD1-2, GOLD3 and GOLD4 patients (H = 20.52, P < 0.0001; H = 19.57, P < 0.0001; H = 6.83, P = 0.033; H = 13.98, P = 0.001; H = 6.56, P = 0.038). The difference between GOLD1-2 and GOLD3 groups for FVC and FEV1 (P < 0.05), and between GOLD1-2 and GOLD4 groups (P < 0.05); R5 was statistically significantly different between GOLD3 and GOLD4 groups (P = 0.042); R5-R20 was statistically significantly different between GOLD1-2 and GOLD4 groups (P = 0.001), between GOLD3 and GOLD4 groups (P = 0.048); X5 was statistically significantly different between GOLD1-2 and GOLD3 (P = 0.032), nevertheless, between GOLD1-2 and GOLD4 was not (P > 0.05), between GOLD3 and GOLD3 was not (P > 0.05). Moreover, the relative between-visit variability (SDbv) and COV for IOS parameters were higher than for spirometry in different stages of COPD.

**Table 3:** Long-term variability of spirometry and IOS parameters in degree of airway obstruction of stable COPD

	COPD-All (N = 73)	COPD-GOLD1-2 (N = 23)	COPD-GOLD3 (N = 31)	COPD-GOLD4 (N = 19)
SDbv				
FVC, L	0.25 (0.16–0.35)	0.17 (0.07–0.24) ^b^	0.29 (0.17–0.36)	0.30 (0.22–0.45)
FEV1, L	0.15 (0.09–0.22)	0.12 (0.06–0.17)	0.17 (0.10–0.23)	0.14 (0.10–0.20)
MEF 50, L/s	0.10 (0.06–0.19)	0.15 (0.07–0.22)	0.11 (0.05–0.19)	0.09 (0.05–0.13)
MEF 25, L/s	0.04 (0.02–0.08)	0.05 (0.03–0.08)	0.04 (0.02–0.05)	0.04 (0.02–0.06)
MMEF 75/25, L/s	0.08 (0.05–0.13)	0.11 (0.07–0.13)	0.07 (0.04–0.13)	0.07 (0.05–0.10)
R5, cmH_2_O/(L/s)	0.86 (0.52–1.28)	0.66 (0.45–1.12)	1.18 (0.86–1.49) ^c^	0.59 (2.27–0.99)
R20, cmH_2_O/(L/s)	0.40 (0.29–0.61)	0.39 (0.27–0.62)	0.49 (0.37–0.82)	0.33 (0.30–0.48)
R5-R20, cmH_2_O/(L/s)	0.71 (0.45–1.16)	0.58 (0.42–0.92)	0.95 (0.57–1.27)	0.51 (0.42–1.00)
AX, cmH_2_O/L	12.33 (6.88–19.30)	5.72 (3.29–10.29) ^a b^	16.48 (11.97–24.07)	14.23 (8.31–19.78)
Freq, 1/s	3.88 (2.33–6.59)	3.13 (1.94–5.07)	8.65 (6.54–14.83)	8.05 (4.18–11.47)
X5, cmH_2_O/(L/s)	1.46 (0.62–2.47)	0.47 (0.36–1.18) ^a b^	1.93 (0.99–2.81)	1.93 (1.20–2.70)
COV				
FVC, L	11% (6–16%)	6% (3–10%) ^a b^	12% (9–15%)	15% (10–21%)
FEV1, L	15% (8–20%)	7% (3–15%) ^a b^	17% (10–23)	17% (12–26%)
MEF 50, L/s	20% (10–31%)	17% (8–22%)	24% (11–38%)	24% (16–35%)
MEF 25, L/s	18% (11–28%)	16% (10–30%)	16% (12–27%)	23% (12–29%)
MMEF 75/25, L/s	17% (12–25%)	17% (12–19%)	21% (11–32%)	20% (16–34%)
R5, cmH_2_O/(L/s)	15% (10–21%)	17% (10–21%)	17% (12–25%) c	10% (3–18%)
R20, cmH_2_O/(L/s)	13% (10–19%)	12% (10–17%)	16% (11–22%)	13% (9–16%)
R5-R20, cmH_2_O/(L/s)	29% (20–45%)	45% (26–59%)	32% (22–43%) ^b c^	21% (9–29%)
AX, cmH_2_O/L	37% (24–56%)	39% (24–62%)	41% (24–60%)	32% (18–48%)
Freq, 1/s	15% (9–23%)	16% (9–25%)	16% (11–25%)	13% (7–18%)
X5, cmH_2_O/(L/s)	37% (22–52%)	23% (17–47%) ^a^	43% (27–66%)	31% (27–45%)
ICC				
FVC, L	0.96	0.98	0.92	0.79
FEV1, L	0.94	0.96	0.79	0.68
MEF 50, L/s	0.94	0.93	0.76	0.66
MEF 25, L/s	0.84	0.88	0.34	0.37
MMEF 75/25, L/s	0.94	0.95	0.75	0.67
R5, cmH_2_O/(L/s)	0.90	0.85	0.90	0.90
R20, cmH_2_O/(L/s)	0.84	0.79	0.83	0.85
R5-R20, cmH_2_O/(L/s)	0.84	0.77	0.80	0.84
AX, cmH_2_O/L	0.85	0.77	0.81	0.73
Freq, 1/s	0.78	0.71	0.65	0.71
X5, cmH_2_O/(L/s)	0.78	0.80	0.75	0.34
COR				
FVC, L	0.71 (25%)	0.64 (19%)	0.72 (35%)	0.75 (50%)
FEV1, L	0.45 (23%)	0.44 (21%)	0.49 (43%)	0.38 (49%)
MEF 50, L/s	0.40 (26%)	0.50 (29%)	0.39 (49%)	0.27 (54%)
MEF 25, L/s	0.18 (45%)	0.20 (39%)	0.18 (83%)	0.15 (83%)
MMEF 75/25, L/s	0.31 (28%)	0.37 (29%)	0.31 (51%)	0.21 (58%)
R5, cmH_2_O/(L/s)	2.73 (35%)	2.42 (44%)	3.21 (36%)	2.17 (34%)
R20, cmH2O/(L/s)	1.62 (47%)	1.38 (50%)	1.97 (50%)	1.13 (42%)
R5-R20, cmH_2_O/(L/s)	2.54 (46%)	2.33 (60%)	2.92 (51%)	2.17 (47%)
AX, cmH_2_O/L	44.35 (42%)	29.76 (63%)	49.94 (45%)	49.16 (53%)
Freq, 1/s	15.05 (50%)	12.27 (56%)	17.21 (60%)	13.00 (52%)
X5, cmH_2_O/(L/s)	5.08 (47%)	2.78 (56%)	5.59 (46%)	5.89 (74%)

Kruskal Wallis test was used to compare with multiple groups, post hoc comparison was analyzed by Bonferroni. ^a^P < 0.05 COPD-GOLD1-2 versus COPD-GOLD3; ^b^P < 0.05 COPD-GOLD1-2 versus COPD-GOLD4; ^c^P < 0.05 COPD-GOLD3 versus COPD-GOLD4. See Table 1 and Table 2 legend for expansion of abbreviations

It is exciting that the higher the COPD stage the lower numerical values of ICC for spirometry parameters. Nevertheless, the ICC values of IOS resistance parameters were relatively stable and significant, and were higher than the ICC values of spirometry parameters in GOLD3 and GOLD4 stages. The ICC values of IOS reactance parameters were relatively low. Higher COPD stage had higher COR values of spirometry parameters. The COR values of IOS resistance parameters were relatively stable and lower than those of spirometry parameters in GOLD3 and GOLD4 stage, the higher the COPD stage, the lower COR values of IOS resistance parameters, the COR values of IOS reactance parameters were relatively stable.

Associations between long-term variability and degree of airway obstruction of stable COPD.

**Fig. 2:**
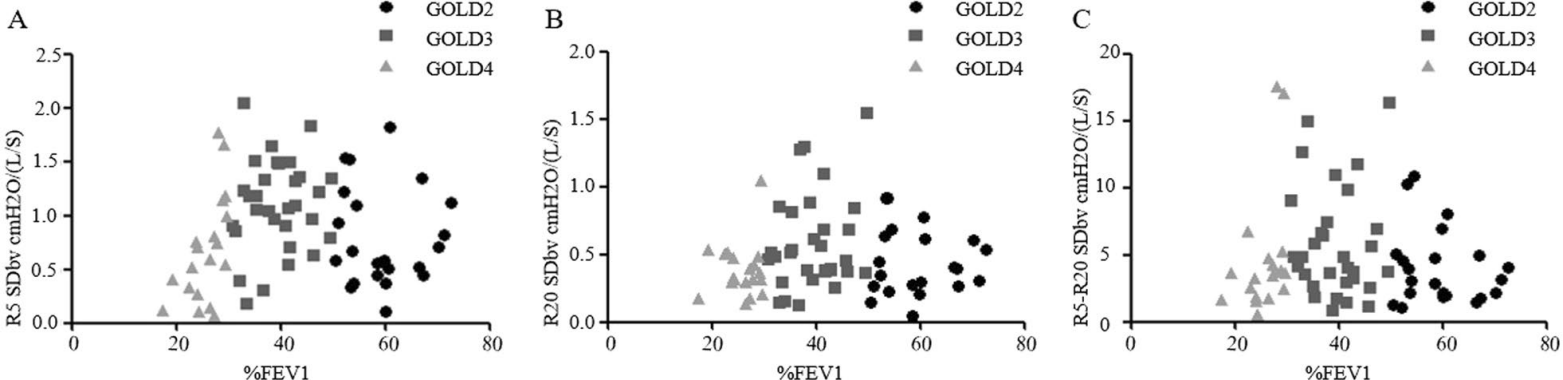
Relationships between spirometry and IOS parameters in the three subject groups. Spearman correlation coefficients for non-parametric data was used. **A** %FEV1 and R5 SDbv (GOLD2 r_s_ = − 0.06, P > 0.05; GOLD3 r_s_ = 0.12, P > 0.05; GOLD4 r_s_ = 0.61, P < 0.05), **B** %FEV1 and R20 SDbv (GOLD2 r_s_ = 0.09, P > 0.05; GOLD3 r_s_ = 0.08, P > 0.05; GOLD4 r_s_ = − 0.04, P > 0.05), **C** %FEV1 and R5-R20 SDbv (GOLD2 r_s_ = − 0.07, P > 0.05; GOLD3 r_s_ = − 0.08, P > 0.05; GOLD4 r_s_ = 0.42, P > 0.05). %FEV1 = FEV1 as a percentage of predicted. See Table 1 and Table 2 legend for expansion of abbreviations

Relationships between airway obstruction and IOS resistance parameters in the three subject groups were shown in Fig. 2. As there was only one case of GOLD1 and its value was large, this case was not analyzed. SDbv of R5 was correlated with %FEV1 (FEV1 as a percentage of predicted) in GOLD4 (r_s_ = 0.61, P < 0.05), not correlated with %FEV1 in GOLD2 and GOLD3(r_s_ = 0.09, P > 0.05; GOLD3 r_s_ = 0.08, P > 0.05) (Fig. 2A); SDbv of R20 and R5-R20 was not correlated with %FEV1 in GOLD stages (P > 0.05) (Fig. 2B, C).

The predictors of long-term variability of the IOS parameters were determined by multiple regression analysis (Table 4). Potential predictors include %FEV1, mean IOS parameter, Age and BMI. %FEV1 was potential predictor for long-term variability of R5 and R5-R20 (t = 2.90, P = 0.005; t = 2.44, P = 0.017), its mean value was a potential predictor for long-term variability of R5, R20 and R5-R20 (P < 0.05). Age and BMI were not potential predictors for long-term variability of IOS parameters.

**Table 4:** Multiple regression analyses to determine long-term variability and airflow obstruction

	B	SE	β-Coefficient	t	P value
Predictors of long-term variability of log R5					
%FEV1	0.007	0.003	0.347	2.899	0.005
Mean R5	0.059	0.020	0.344	2.914	0.005
Age	− 0.003	0.004	− 0.075	− 0.650	0.518
BMI	− 0.005	0.010	− 0.056	− 0.498	0.620
Predictors of long-term variability of log R20					
%FEV1	0.000	0.002	− 0.006	− 0.051	0.959
Mean R20	0.118	0.037	0.357	3.158	0.002
Age	0.001	0.003	0.053	0.478	0.634
BMI	0.012	0.008	0.168	1.476	0.144
Predictors of long-term variability of log R5-R20					
%FEV1	0.007	0.003	0.303	2.441	0.017
Mean R5-20	0.108	0.031	0.437	3.509	0.001
Age	0.002	0.004	0.057	0.500	0.618
BMI	− 0.013	0.010	− 0.144	− 1.304	0.196

Multiple regression analysis was performed using SPSS 22.0. See Table 1 and Table 2 legend for expansion of abbreviations

## Discussion

We measured long-term variability of IOS parameters in stable COPD and asthma in this study. According to the demographic data and the baseline lung function test results of the COPD and asthma patients, although asthma [(49.86 ± 14.26) years] patients were younger than COPD [(62.68 ± 9.57) years] patients, the spirometry parameters in COPD were lower compare to asthma (P < 0.0001), the COPD patients had higher R5, R5-R20, AX and more negative X5, except for R20 and Freq, compared to the asthma patients (P < 0.0001). These findings suggest that there were differences in pulmonary ventilation function and airway obstruction between COPD and asthma.

As airway obstruction increases with disease progression and airway remodeling, characteristic changes in resistance and reactance are more pronounced. Particularly, resistance spectrum increases in direction of low frequencies. At the same time overall resistance increases but more so at low frequencies than at high frequencies [16, 17]. Thus, when we compared the long-term variability of spirometry and IOS parameters in COPD and asthma, we found that SDbv of FVC, FEV1, MEF 50, MEF 25, MMEF75/25, R5, R5-R20, AX, Freq and X5 were statistically significantly different in COPD and asthma, SDbv of FVC, FEV1, R5, R5-R20, AX, Freq and X5 in COPD were higher compare to asthma, SDbv of MEF50, MEF25 and MMEF75/25 in COPD were lower compare to asthma. SDbv of R20 had no difference between COPD and asthma. COV for FVC, FEV1, MEF50, MMEF75/25, R5-R20 and X5 were statistically significantly different between COPD and asthma. However, R5, R20, AX and Freq were not. COV of FVC, FEV1, MEF50, MMEF75/25, and X5 in COPD were higher than those in asthma. SDbv and COV of FVC, FEV1, MEF 50, MEF 25, MMEF75/25 in IOS parameters were higher compare to spirometry. These results indicate that long-term variability of IOS parameters in COPD was higher than in asthma, and long-term variability in IOS parameters was higher than spirometry in COPD and asthma.

COR values were less than 66% in COPD and asthma, except for Freq (69%) and X5(80%) in asthma with the relative CORs for IOS being more variable than for spirometry. However, the repeatability of IOS parameters, apart from Freq and X5, were high (> 0.80). We are not sure if this has any relationship to the difference of distal airway distensibility in the two conditions. These results suggest that despite there being higher long-term variability in IOS measurements than spirometry, IOS is still highly repeatable and stable. The high variability may be due to different baseline characteristics, the airway caliber and elastic characteristics of respiratory system, which fluctuated with time [18, 19].

The current gold standard to assess airway limitations is spirometry. However, performing an optimal spirometry always requires good patient cooperation. Additionally, repeated forced breathing causes changes in bronchial motor tension, false positive results occur often. Studies on the quality of spirometry in elderly patients have shown that only 30% of patients are able to perform a spirometry that meets the quality standards of the European Respiratory Society/American Thoracic Society[20, 21]. Furthermore, FEV1 cannot fully assess small airway abnormalities. Thus, MEF50, MEF25 and MMEF75/25 have been studied as markers of small airway obstruction but are highly variable due to atmospheric airway obstruction [22]. We found that between-visit variability (SDbv) of FVC, R5, AX, X5 were statistical difference between GOLD1-2, GOLD3 and GOLD4 groups. Small airways assessed by IOS parameters including X5 and AX correlate more strongly with clinical symptoms than with spirometry [23]. Between-visit variability relative to the mean COV for FVC, FEV1, R5, R5-R20 and X5 were statistically significantly different in GOLD1-2, GOLD3 and GOLD4 patients. The values of SDbv and COV for IOS parameters were higher than for spirometry in different stages of COPD. The higher the COPD stage, the lower ICC values of spirometry parameters (FVC, FEV1, MEF50, MEF25 and MEF75/25), the ICC values were less than 0.8 in GOLD3 and GOLD4. The lower ICC values of FVC, FEV1 perhaps were because COPD patients with poorer lung function are less able to cooperate. Nevertheless, the ICC values (> 0.8) of IOS resistance parameters were relatively stable and high. Similarly, the higher the COPD stage, the higher COR values of spirometry parameters, but the COR values of IOS parameters were relatively stable. The results show that long-term variability of spirometry parameters (FVC, FEV1, MEF50, MEF25 and MEF75/25) was higher, repeatability was lower than IOS parameters in different GOLD stages. Additionally, the higher the stage, the worse the repeatability. In COPD it is well established that the changes in R5, R20 correlate well with GOLD1 to GOLD4 severity and our results tend to corroborate this. IOS resistance parameters remain relative stable and reproducible over time compare to IOS reactance parameters. Reactance is comprised of both inertance and elastance. Diseases (for example: interstitial lung diseases) that influence the elasticity of the lung will increase capacitance negatively and X5 will be more negative. Values of reactance parameters are affected by age and weight, increase of age and weight will determine a less negative reactance. Moreover, reactance is affected by the heterogeneous distribution of airway calibres and lung compliances [2, 24]. These factors may lead to high variability and poor repeatability of reactance. So, IOS resistance parameters can be used as a routine adjunct to lung function test.

The short-term variability in IOS parameters is known, particularly within-day, day-to-day or week-to-week repeatability of resistance (Rrs) and reactance (Xrs) with high ICC values (> 0.80) [25–27]. It is the first study to relate measurement of long-term variability to airflow obstruction. Previous studies have largely assessed the within session repeatability and variability of resistance [28, 29]. However, these studies may not be applicable to clinical settings, where patients in clinically stable stage of disease are often examined several months apart. In this study, repeatability of IOS measurements between clinical visits was a representation of the real-world behavior of these parameters. The median (IQR) time between first and third visit was 6.1 (3.4–16.6) months in COPD and 4.9 (4.0–12.6) months in asthma (Table 1). Only one study conducted a long-term variability analysis of IOS parameters in stable COPD and asthma [10]. They only performed a long-term variability analysis at consecutive three follow-ups. Based on what is already known, with this study our research reveals: (1) Our sample size was much larger; (2) Long-term variability between IOS parameters and spirometry in COPD, COPD-GOLD stages, and asthma was further compared; (3) It was also the first study to determine the relationship between long-term variability and airflow obstruction in COPD.

Significant correlations at baseline between FEV1 and R5 (P < 0.05) but not with R20 has been reported and during 1-year follow up. The changes in R5 and R20 did not significantly correlate with FEV1. Additionally, the R20 is unrelated to the severity of airflow obstruction in patients with COPD [30]. We found that during the first three visits, SDbv of R5 correlates with %FEV1 in GOLD4 (r_s_ = 0.61, P < 0.05). Additionally, multiple regression analyses indicated that %FEV1 is potential predictor for long-term variability of R5 and R5-R20 (t = 2.90, P = 0.005; t = 2.44, P = 0.017). Their mean values are potential predictor for long-term variability of R5, R20 and R5-R20 (P < 0.05). The severity of airflow obstruction significantly was correlate with long-term variability of R5 and R5-R20, not with long-term variability of R20.

One potential limitation of this study was that the groups were not matched by sex and age in different diseases and GOLD stages. There was one female in GOLD1, 20 male and 2 female in GOLD2, 25 male and 6 female in GOLD3, 18 male and 1 female in GOLD4, 58 male and 61 female in asthma. There is long-term disease progression in stable diseases [31], our maximum follow-up period was 22 months. Longer follow-up period to assess long-term variability would perhaps lend more credibility to future studies.

## Conclusions

This study revealed that IOS resistance parameters have high long-term repeatability as shown by high ICC values (P > 0.80) in asthma and COPD with lower ICC values < 0.8 in GOLD3 and GOLD4. Higher long-term variability seen in COPD was related to airflow obstruction. These findings help determine thresholds for MCIDs. IOS can support routine lung function testing to monitor disease progression, disease activity, disease progression or treatment response over a long period of time. IOS will not replace spirometry anytime soon as the two tests measure very different things. However, when IOS is used as a complimentary test to spirometry for long term follow up, proves to be clinically very useful.

## Data Availability

All the related data are presented in the manuscript.

## Abbreviations

COPD: Chronic obstructive pulmonary disease
IOS: Impulse oscillometry
SDbv: The standard deviation
COV: The coefficient of variation
ICC: Intraclass correlation coefficient
COR: The coefficient of repeatability
FOT: Forced oscillation technique
R5: R at 5 Hz
R20: R at 20 Hz
X5: Xrs at 5 Hz
Freq: Resonant frequency
AX: A low-frequency reactance area
FEV1: Forced expiratory volume in 1 s
MCID: The minimal clinically important difference
PFT: Pulmonary function tests
GOLD: The Global Initiative for Chronic Obstructive Lung Disease
FVC: Forced vital capacity
pMV: Percentage of close to maximal variation
BMI: Body mass index
MEF: Maximal expiratory flow
MMEF: Maximum mid expiratory flow
Rrs: Resistance
Xrs: Reactance
IQR: The median
%FEV1: FEV1 as a percentage of predicted
